# Investigating the association between alcohol intake and male reproductive function: A current meta-analysis

**DOI:** 10.1016/j.heliyon.2023.e15723

**Published:** 2023-04-24

**Authors:** Tung Nguyen-Thanh, Ai-Phuong Hoang-Thi, Dang Thi Anh Thu

**Affiliations:** a Faculty of Basic Science, Hue University of Medicine and Pharmacy, Hue University, Hue, 49000, Viet Nam; b Institute of Biomedicine, Hue University of Medicine and Pharmacy, Hue University, Hue, 49000, Viet Nam; c Faculty of Public Health, Hue University of Medicine and Pharmacy, Hue University, Hue, 49000, Viet Nam

**Keywords:** Alcohol consumption, Heavy drinkers, Sperm parameters, Testosterone, Antioxidant, Sex hormones, Sperm DNA fragmentation

## Abstract

**Background:**

Alcohol use and alcohol-related health problems are on the rise in developing countries. This meta-analysis was conducted to determine the effects of alcohol consumption on human male reproductive function through semen parameters, antioxidants in semen, sperm DNA fragmentation, and sex hormones.

**Methods:**

Studies regarding the effects of alcohol consumption on male reproductive function were searched on databases. Based on the random-effects model, STATA software was used to analyze and synthesize the selected studies. Alcoholics, moderate alcoholics, heavy alcoholics, and no alcoholics values were compared using the standard mean difference. Publications were assessed for publication bias by the Egger test.

**Result:**

Forty studies were selected from databases examining the effect of alcohol consumption on male reproductive health in 23,258 people on five continents of the world. The meta-analysis revealed that alcohol intake reduced semen volume during each ejaculation (SMD = −0.51; 95% CI −0.77, −0.25). However, there were no significant associations with other semen indicators such as density, mobility, and normal and abnormal sperm count from this analysis. In addition, drinking alcohol lowered antioxidant enzymes in semen (SMD = −7.93; 95% CI −12.59, −3.28) but had no effect on sperm DNA fragmentation. Finally, the results showed a decrease in general testosterone levels (SMD = −1.60; 95% CI −2.05, −1.15), Follicle Stimulating Hormone (SMD = −0.47; 95% CI −0.88, −0.05), Luteinizing Hormone (SMD = −1.35; 95% CI −1.86, −0.83), but no effect in other sex hormones named as estradiol, Inhibin B and Sex Hormone-Binding Globulin. Furthermore, when analyzing subgroups at different drinking levels, the results showed that the moderate alcoholic group (less than 7 units/week) had no change in the semen index. Meanwhile, the group of heavy alcoholics (more than 7 units/week) harmed the semen index and sex hormones, especially by increasing estradiol.

**Conclusion:**

There is evidence that alcohol consumption affected semen volume and antioxidant, reproductive hormones thus negatively affecting male reproductive function. This study might be necessary to make recommendations regarding alcohol consumption for men.

## 1 Introduction

The use of alcoholic beverages in daily life is considered a cultural feature of many countries in the world. They were used in several religions because they bring excitement and addiction to consumers [1]. According to a recent WHO study, one of the alarming current issues is that while alcohol use is declining in developed countries, it is increasing in developing countries [[2], [3], [4]]. From 1993 to 2000, alcohol consumption per capita among Vietnamese adults increased by roughly 2.5 times [5]. Excessive or long-term alcohol consumption has negative consequences not only for the person who consumes it but also for the family and society [[6], [7], [8], [9]]. Reports suggest that unhealthy alcohol use affects nearly every organ system [[10], [11], [12], [13], [14]].

Alcohol consumption affects the entire hypothalamic-pituitary-gonadal region of the male reproductive system and disrupted the production of Gonadotropin-Releasing Hormone (GnRH), Follicle Stimulating Hormone (FSH), and Luteinizing Hormone (LH), the natural oestradiol levels due to changes in free testosterone, impaired function of Leydig and Sertoli cells, resulting in reduced sperm quality to normal morphology and sperm maturation [[15], [16], [17]]. Consumption of alcohol with a high concentration and frequency has led to negative effects on the concentration of sperm; normal morphology of spermatozoa; total sperm count, reduced testosterone and SHBG, and increased serum testosterone levels [18,19]. Scientific evidence shows that it causes impotence, infertility, and spermatogenic arrest (Pajarinen JT and Karhunen 1994, [[20], [21], [22], [23], [24], [25]]. Many studies showed negative impacts based on evidence that drinking alcohol leads to an increase in enzymatic antioxidants, sperm DNA damage [26], and high levels of DNA fragmentation [27]. However, several reports give the opposite findings. Barratt et al. have shown that moderate alcohol consumption did not affect semen quality despite higher testosterone levels [28]. The reports of Teijon et al. and Hansen et al. show no statistically significant difference in semen parameters between the alcoholics and no-alcoholics [29] and no association between alcohol consumption and sperm quality [15]. Moreover, a meta-analytical conducted by Ying Li et al. found drinking alcohol did not affect sperm parameters [20].

For a more general and accurate assessment of existing information, we conducted a meta-analysis of published literature that assessed the effects of alcohol intake and male reproductive function. This report shows a general opinion on drinking alcohol and parameters related to male reproductive quality and provides recommendations on alcohol consumption with concentrations and levels defined.

## 2 Materials and methods

### 2.1 Search strategy and identification of relevant studies

Research subjects are scientific publications on databases that study the associations between alcohol consumption and male reproductive health parameters such as semen parameters, antioxidant enzymes, sperm DNA fragmentation, and sex hormone levels. The studies were searched from MEDLINE, EMBASE databases, and scientific websites through keywords: “alcohol consumption”, “DNA fragmentation”, “semen quality”, “semen parameter”, “normal morphology”, “reproductive hormones”, “alcohol”, “motility”, “male fertility”, “testosterone”, “estradiol”, “inhibin B”, “oxidative damage”, “semen quality”, “sperm abnormalities”, “alcohol abuse”, “male fertility”, “enzymatic antioxidant activity”.

The selection criteria include articles in English, having full text, and providing data of interest. Exclusion criteria reviewed articles that provided no data or only in the form of graphs, cell, and animal studies. Studies were selected when they: (a) evaluated the association between alcohol consumption and male reproductive health parameters; (b) defined drinking levels; (c) effect of alcohol consumption on sex hormones; (d) represented original data; (e) used a cohort study design, and (f) were written in English. Papers were excluded when they: (a) described a review, case report, or conference abstract; (b) did not contain original data; (c) did not provide data.

### 2.2 Collect data for analysis (Data extraction)

The studies extracted data regarding the effect of alcohol on indicators of male reproductive function such as semen volume (mL), concentration (10^6^ sperm/mL), ability semen motility (%), normal morphology, abnormal morphology, and sperm defects, sperm DNA fragmentation (SDF) (%), testosterone (ng/mL), β-estradiol (pg/mL), Inhibin B (pg/mL), sex hormone-binding globulin (SHBG) (nmol/L), Follicle Stimulating Hormone (FSH) (mUI/mL), Luteinizing Hormone (LH) (mUI/mL). Metric values were described as mean and standard deviation or median and IQR. If data are provided as median and IQR, they were converted to mean and standard deviation before proceeding with the meta-analysis [[30], [31], [32]].

### 2.3 Statistical analysis

A meta-analysis of the studies was done using the statistical analysis software STATA 15 based on the random effects model (Random Effects Model). Heterogeneity between studies was assessed through the heterogeneity index I-squared. The values groups were compared using the standard mean difference (SMD). Publications are evaluated for publication bias using the Egger test.

### 2.4 Subgroup analysis

We employed subgroup analysis by drinking levels definition (according to alcohol concentration and frequency consumption). For that there are three groups of people based on their drinking habits: no-alcoholics if they drink less than 1 unit of alcohol a week; moderate alcoholics if they use at least 1 unit/week to 7 units/week; and heavy alcoholics when they take more than 7 units of alcohol/week. One unit of alcohol is equivalent to 7.9 g of ethanol or 10 mL of ethanol contained in an oral solution [33]. Thus, 1 unit of alcohol is equivalent to 3/4 bottles/cans of 330 mL beer (5% alcohol); 1 cup of draft beer 330 mL; 1 glass of wine 100 mL (13.5% alcohol); or 1 cup of spirits 30 mL (40% alcohol).

## 3 Results

### 3.1 Characterization of eligible studies

**Fig. 1:**
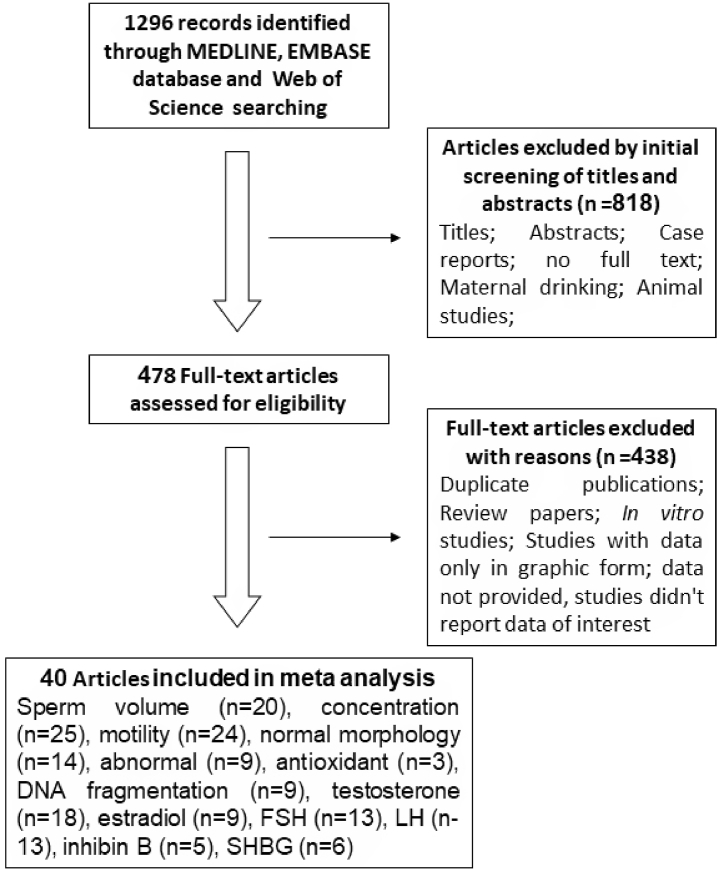
The selection process of papers for the meta-analysis study.

Studies were searched on scientific databases MEDLINE, EMBASE, and scientific websites. The process of searching and filtering the studies for the meta-analysis is shown in Fig. 1. The results of 1296 studies were found, of which 818 were omitted because the title and abstract did not match the selection criteria to conduct the review. The remaining 478 full-text articles continued to be evaluated. Of these, 438 articles were excluded for reasons such as duplicate content, some reviews, animal studies, *in vitro* studies, studies with graphical data only, and articles that had not provided complete data. In the end, we obtained 40 studies that were eligible for inclusion in the systematic review, of which 20 reported on alcohol consumption and semen volume (mL), 25 articles included data on sperm density (million/mL), 24 articles had information on sperm motility of (percentage of motile sperm), 14 articles show results on sperm morphology. The remaining studies involved alcohol use and other indicators such as DNA fragmentation (9 studies), testosterone (18 studies), estradiol (9 studies), FSH (13 studies), LH (13 studies), inhibit B (5 studies), SHBG (6 studies) [15,18,26,[34], [35], [36], [37], [38], [39], [40], [41], [42], [43], [44], [45], [46], [47], [48], [49], [50], [51], [52], [53], [54], [55], [56], [57], [58], [59], [60], [61], [62], [63], [64], [65], [66], [67], [68], [69], [70]] (Fig. 1 and Table 1).

**Table 1:** Characteristics of included studies assessing an association between alcohol intake and male reproductive function.

Study	Year	Country	Age	Participation	Sample size					Research index	Main outcome
					n	Drinking Levels Defined					
						No-alcoholics	Moderate alcoholics	Heavy alcoholics	Alcoholics		
Anifandis	2014	Greece	37.43 ± 0.3	Men who were seeking semen analysis for fertility purposes in IVF Unit	207	124	58	25	83	V, C, M, DF	Heavy alcohol consumption may impair sperm volume, concentration, and sperm DNA fragmentation
Chia	1998	Singapore	33.2 ± 5.3	Fertile men	243	146			97	V, C, M, NM,	Social alcoholic consumption did not appear to affect sperm quality
Condorelli	2014	Italy	34.0 ± 6.0 31.0 ± 8.0	Fertile and infertile men	76	0	40	36	76	V, C, M, NM, T, E, FSH, LH	‘Daily drinkers’ have semen quality, testicular pathology, and hormonal characteristics significantly worse
Eskenazi	2003	USA	44.5	Non-smoking men without known fertility problems	97	34			63	V, C, M,	Semen volume and sperm motility decreased continuously
Goverde	1995	Netherlands		Men with poor semen quality	47	20			27	V, C, M, NM,	Alcohol consumption may further decrease an already low percentage of sperm with normal morphology
Hansen	2012	Denmark		Young Danish men	345	54	84	109	291	V, C, M, NM, DF, T, E, F, L, I, S	Alcohol intake and a hormonal shift towards a higher estradiol/testosterone ratio
Hart	2015	Australia	20 ± 0.5	Fertile men	887	120	463	304	767	C, T, E, F, L, I,	Alcohol intake was not associated with any significant semen variables or circulating reproductive hormones
Jensen *(a)*	2014	Denmark	18–28	Young Danish men attending military service from 2008 to 2012	1206	176	680	92	1030	V, C, M, NM, T, E, F, L, I, S	Alcohol consumption was also linked to changes in testosterone and SHBG levels.
Jensen (b1)	2014	Europe	18–28	Fertile young men	6472	1133		2872	5339	V, C, M, NM, T, F, LH, I, S	Alcohol consumption is not related to semen quality
Jensen (b2)	2014	Europe and USA	18–45	Fertile men	1872	560		429	1312	V, C, M, NM, T, F, LH, I, S	Alcohol consumption is not related to semen quality
Joo	2012	Korea	39.5 ± 2.6 35.4 ± 1.2	Fertile men and nonsmokers	13	8		5	13	V,C,M, NM,	Alcohol consumption was associated with increased numbers of morphologically abnormal sperm.
Kumar	2014	India	31.91 ± 0.37	Oligozoospermia	63	54			9	C, M, NM,	Alcohol use might be attributed to the risk of declining semen quality
Lopez Teijon	2007	Spain	32.6 ± 6.0	Volunteers from the province of Barcelona	967	440			527	C, M, NM	The consumption of alcohol did not have a significant effect on semen quality
Martini	2004	Argentina		Semen samples (one per patient) with toxic habits	3430	3194			236	V, C, M, NM	Alcohol consumption did not alter the seminal parameters
Muthusami	2005	India	36.6 ± 5.7 35.0 ± 6.1	Nonsmoking alcoholics - normal healthy persons	96	30			66	V, C, M, NM, AM, T, E, F, Lh, I, S,	Alcohol consumption has a detrimental effect on male reproductive hormones and semen quality
Kucheria	1985	India	25–42	Alcohol Dependence Syndrome – control case	30	10			20	V, C, M, AM, T, F, L,	Alcohol consumption for a considerable period affects spermatogenesis, spermiogenesis and causes oligozoospermia
Brzek	1987	Czechoslovakia	35,7–33.1	Alcoholics and control group	235	135			100	V, C, M	Patients with alcohol abuse had defective stereograms
Komiya	2014	Japan	40,7 ± 4,1	Male Japanese patients with infertility	54	27			27	DF,	Chronic alcohol use increased the SDF
Keskin	2016	Turkey	33.0 ± 5.43	Infertile men who use alcohol	356	256	81	19	100	V, C, M, NM, AM	There was no significant difference in any of the parameters
Wdowiak	2016	Poland	35.0 ± 4.82	Couples who had been diagnosed with infertility	499	345			154	DF,	The percentage of sperm cells with nuclear DNA strand breaks was significantly higher in men with risky alcohol consumption
Ricci	2018	Italy	39.3 ± 5.2	Couples who had been diagnosed with infertility	323	31	195	97	292	V, C, M	Moderate alcohol intake appears positively associated with semen quality in male partners of infertile couples undergoing ARTs
Aboulmaouahib	2018	Morocco	39.23 ± 8.87 37.95 ± 7.83	Infertile couples	59	36			23	V, C, M, NM, DF, AO	Alcohol intake had detrimental effects on DNA integrity with the potential adverse effects of OS on fertility.
Schmid	2007	USA	41.8 ± 14.9 47.5 ± 14.5	Non-smokers	80	30			50	DF	Alcohol use affects sperm DNA fragmentation
Varshini	2012	India	21–40	Men who visited the University infertility clinic between 2006 and 2010	504	445			59	DF	Alcoholics had a significantly higher median distribution of TUNEL-positive spermatozoa
Schmid	2012	USA	46.4	Healthy male volunteers	79	31			48	DF	Men with higher dietary and supplement intake of certain micronutrients may produce sperm with less DNA damage
Marshburn	1989	USA		Men couples are seen at the University of North Carolina infertility clinic between 1978 and 1982.	446	338			108	V, C, M, AM,	Ethanol could produce addictive effects on semen parameters.
Wogatzky	2012	Austria	40.4 ± 5.9	Men with undergoing ART	1573	282	1127	164	1291	V, C, M	Using alcohol does not affect sperm
Gautam	2015	India	29.5 ± 5.4 28.5 ± 4.3	Fathers of children with retinoblastoma and controls	56	31			25	DF	ROS and DFI levels in alcohol users were higher as compared to alcohol non-users.
Maneesh	2006	India	29.6 ± 4.2	Male alcohol abusers- healthy male volunteers	101	55			46	T, F, L, AO	Ethanol caused low plasma testosterone in men accompanied by low LH and FSH
Shiels	2009	USA	41.5 ± 0.7	Men C20 years old who participated in the Third National Health and Nutrition Examination Survey	1275	466			809	T, E, S,	Alcohol consumption activity may be associated with concentrations of sex steroid hormones among adult men.
Frias	2002	Spain	20–27	Men with acute alcohol intoxication	23	11			12	T, F, L,	Effect of alcohol on pituitary-gonadal axis hormones in humans
Handa	1997	Japan	50–54	Men who received a preretirement health examination at the Self-Defence Forces Fukuoka Hospital	303	59			224	T, F,	Drinking alcohol can affect estradiol
Kulkarni	2009	India	37.65 ± 6.6 38.30 ± 6.3	Alcohol patients and controls	360	200		160	160	T, F, L, AO	Alcoholic patients displayed significantly low levels of serum testosterone, LH, FSH.
Oldereid	1992	Norway		Men attending our laboratory	239	56			183	C, M, AM,	Alcohol did not appear to have any deleterious effects on sperm quality
Close	1990	USA	32.9	Infertile men	136	82	46	8	54	C, M	Alcohol showed no decrease in sperm count, percentage of motility of oval sperm
Von Der	2002	Finland	20–50	Male residents of Helsinki	84	44			40	T, E,	Alcohol misuse can affect testosterone levels.
Sierksma	2004	Finland	45–64	Middle-aged men, apparently healthy nonsmoking, and moderate alcohol drinkers,	10	5			5		Alcohol consumption can increase testosterone levels
Frias	2000	Spain	13–17	Young adolescents using alcohol	21	10			11	T, E, FSH, LH,	There are many serious effects of alcohol abuse on reproductive function
Iturriaga	1995	Chile	37.3 ± 7.6	Male chronic alcoholics	45	15			30	T, FSH, LH, SHBG,	Alcohol consumption leads to lower FSH and higher SHBG levels
Dai	1981	USA	51 ± 5.4	Men are considered to be free of clinical heart disease	239	33	66	140	206	T	Alcohol intake was not found to be related to testosterone concentrations.

V: Semen volume (mL), C: Concentration (10″6 spermatozoa/mL), M: Progressive motility (%), NM: Normal morphology, AM: Abnormal morphology and sperm defects, AO: Antioxidant, DF: Sperm DNA fragmentation (%), T: Testosterone, E: Estradiol, F: FSH, L: LH, I: Inhibin B, S: SHBG2.

**Fig. 2:**
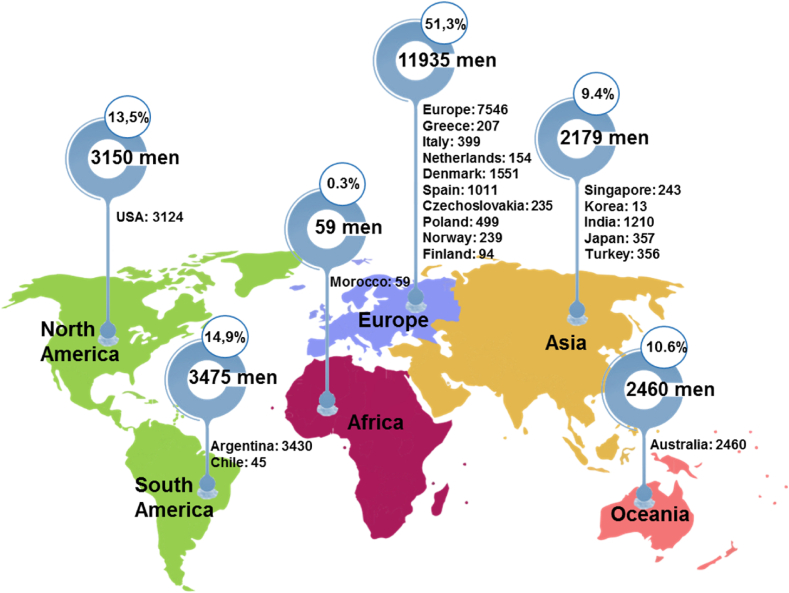
Worldwide distribution of 40 studies with 23,258 men reporting in this meta-analysis.

40 studies published separately over the 37 years from 1981 to 2018 examined the effects of alcohol consumption on the male reproductive health of 23,258 men from 5 continents of the world, of which 11,935 were from Europe (51.3%), 6625 people from America (South America: 14.9%, America North: 13.5%), 2460 people from Australia (10.6%), 2179 people from Asia (9.4%) and 59 people from Africa (0.3%) (Fig. 2).

### 3.2 Association between alcohol consumption and semen parameters

**Fig. 3:**
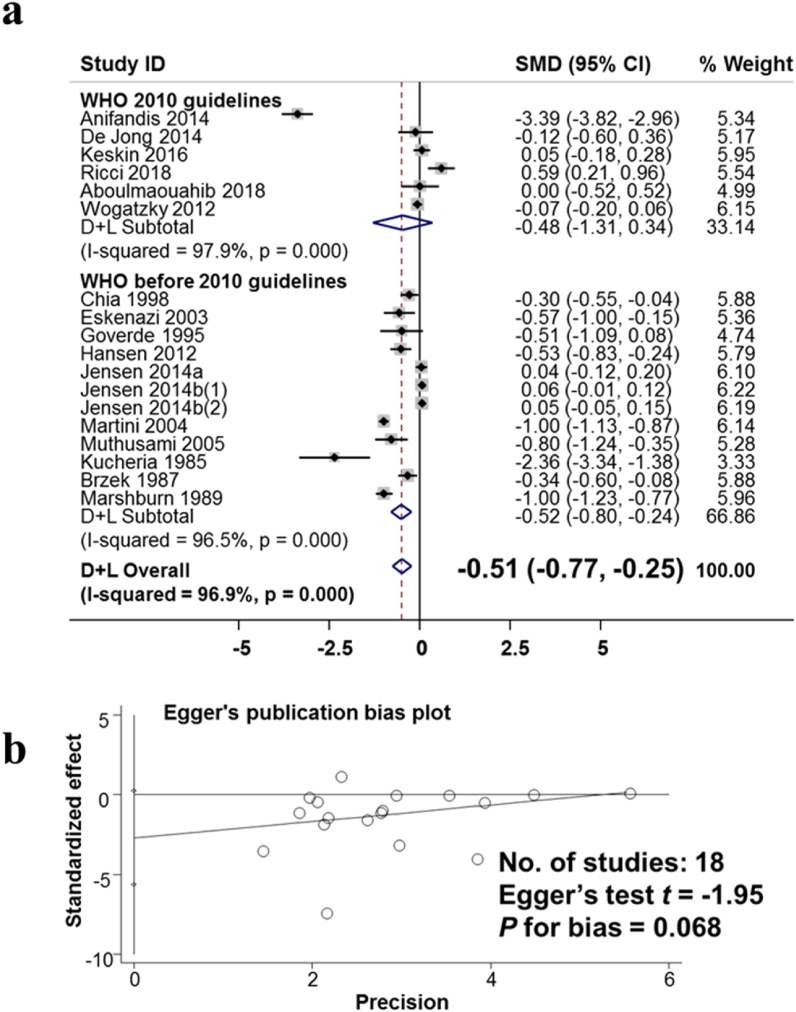
The observed association of alcohol consumption and the decreased semen volume. a. Forest plot for the effect of alcohol consumption and semen volume. b. Evaluating publication bias among studies by Egger publication bias plot.

The effects of alcohol consumption on semen volume were analyzed across 18 studies involving 17,144 male adults are shown in Fig. 3a. The studies were analyzed based on the random-effects model. The difference between studies was large, with the I-squared heterogeneity index being 96.9%. Each study was weighted (%weight) from 3.33 to 6.22. The results of the meta-analysis showed that the standard mean difference (SMD) between the no-alcoholics and alcoholic groups was −0.51 (95% CI: −0.77, −0.25). This result suggests that drinking alcohol reduces the volume of semen in each ejaculation. Fig. 3b presents the results of the assessment of publication bias in publications on the effects of alcohol consumption on semen volume using the Egger test. The results showed no publication bias with p for bias being 0.068.

**Fig. 4:**
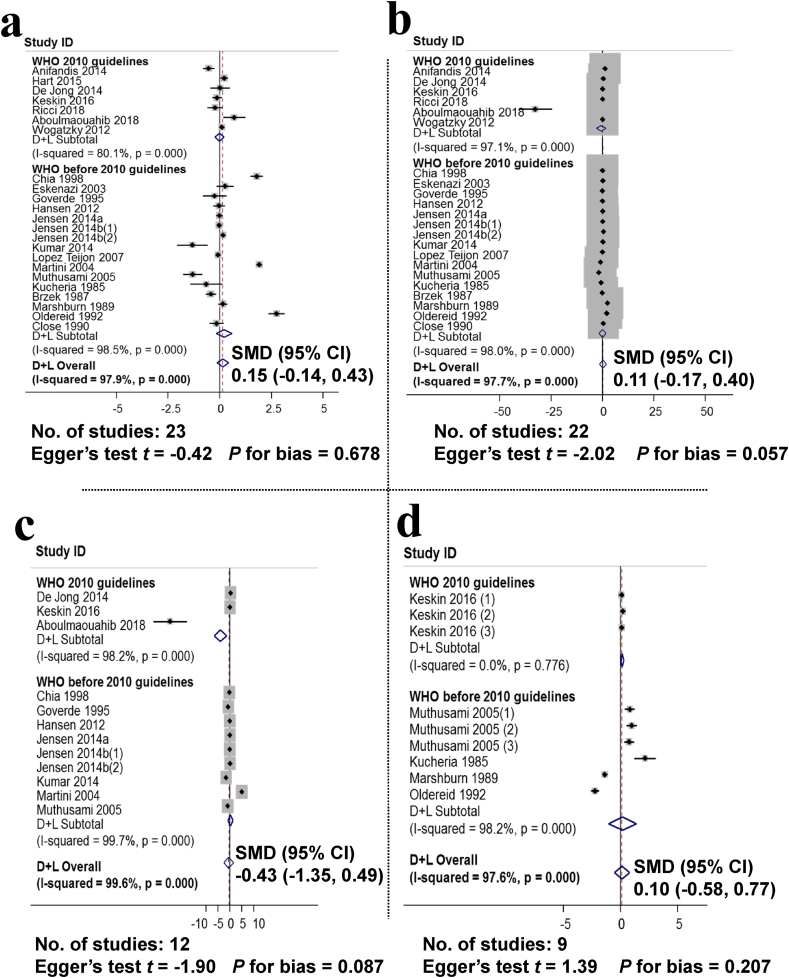
Non-observed associations between alcohol consumption and sperm concentration, motility, normal and abnormal morphology. a. Forest plot for the effect of alcohol consumption and sperm density, assessment of publication bias using the Egger publication bias plot; b. Forest plot for the effect of alcohol consumption and sperm motility and assessment of publication bias using the Egger publication bias plot; c. Forest plot for the effect of alcohol consumption and normal morphology and assessment of publication bias using the Egger publication bias plot; d. Forest plot for the effect of alcohol consumption and abnormal morphology and assessment of publication bias using Egger publication bias plot.

Fig. 4 presents an analysis of the effect of alcohol consumption on sperm density (23 studies, with 19,436 men), motility (22 studies, with 18,549 men), normal morphology (12 studies, with 14,296 men), and abnormal morphology (9 studies, with 2071 men). The results of the analysis showed that the SMD between the no-alcoholics and alcoholics groups was 0.15 (95% CI: −0.14, 0.43), sperm motility 0.11 (95% CI: −0.17, 0.40), SMD of normal sperm morphology is −0.43 (95% CI: −1.35, 0.49). SMD of abnormal sperm morphology was 0.10 (95% CI: −0.58, 0.77). This result shows that drinking alcohol did not significantly affect the parameters of sperm density, motility, normal morphology, and abnormal morphology.

### 3.3 Associations between alcohol consumption and antioxidant enzymes, sperm DNA fragmentation

**Fig. 5:**
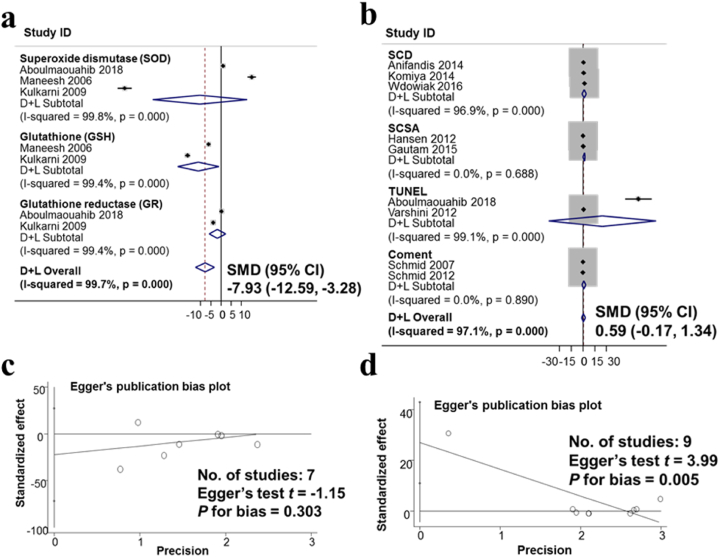
The observed association of the decreased antioxidant enzymes and non-observed association of sperm DNA fragmentation with Alcohol consumption. a. Forest plot for the effect of alcohol consumption and antioxidant enzymes in semen; b. Forest plot for the effect of alcohol consumption and sperm DNA fragmentation; c. Evaluation of publication bias regarding antioxidant enzymes in semen using Egger publication bias plot; d. Evaluation of publication bias about sperm DNA fragmentation using Egger publication bias plot.

The results of an analysis of 1400 men from seven studies on the effects of alcohol consumption on antioxidant enzymes are shown in Fig. 5a. The studies were analyzed based on the random-effects model. The studies have a large variation in research results, the heterogeneity index I-squared heterogeneity was 96.9%. The results of the meta-analysis show that the difference in SMD between the no-alcoholics and alcoholics groups was −7.93 (95% CI: −12.59, −3.28). This result suggests that drinking alcohol reduces antioxidant enzymes in semen. Fig. 5c presents the results of the assessment of publication bias in publications on the impact of alcohol consumption on antioxidant enzymes using the Egger test. The results show no publication bias with p for bias was 0.303.

The effect of alcohol intake on sperm DNA fragmentation in 1883 men from 9 meta-analyzed studies (Fig. 5b). The studies were analyzed based on the random-effects model. The studies have a large variation in research results, the heterogeneity index I-squared heterogeneity was 96.9%, The results of the meta-analysis show that the standard means difference in SMD between the drinking and no-drinking groups was 0.59 (95% CI: −0.17, 1.34). This result shows that drinking alcohol had not to change the sperm DNA fragmentation index. Fig. 5d presents the results of the assessment of publication bias in publications on the impact of alcohol consumption on sperm DNA fragmentation using the Egger test. The results show publication bias with p for bias being 0.005.

### 3.4 Associations between alcohol consumption and sex hormones

**Fig. 6:**
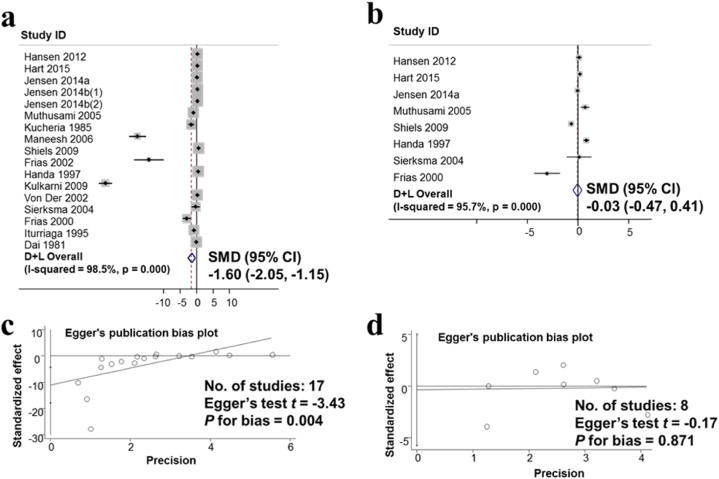
The observed association of the decreased hormone testosterone and non-observed association of hormone Estradiol with Alcohol consumption. a. Forest plot for the effect of alcohol consumption and the hormone testosterone; b. Forest plot for the effect of alcohol consumption and the hormone β-Estradiol; c. Evaluation of publication bias about the hormone testosterone using Egger publication bias plot; d. Evaluation of publication bias using Egger’s publication bias plot.

The results of the study on the effects of alcohol consumption on the hormone testosterone from 17 studies involving 13,373 men were meta-analyzed and presented in Fig. 6a. The studies were analyzed based on the random-effects model. The studies have a large variation in research results, the heterogeneity index I-squared heterogeneity was 96.9%, The results of the meta-analysis show that the standard means difference in SMD between the drinking and non-drinking groups was −1.60 (95% CI: −2.05, −1.15). These results suggest that alcohol consumption lowers the testosterone hormone. Fig. 6c presents the results of the assessment of publication bias in publications on the effects of alcohol consumption on testosterone using the Egger test. The results show publication bias with p for bias was 0.004.

The results of the estradiol hormone survey on 4143 men from 8 studies show that the alcoholic group did not change the estradiol hormone compared with the no-alcoholic group with SMD values of −0.03 (95% CI: −0.47, 0.41) in Fig. 6b. Evaluation of publication bias in publications on the impact of alcohol consumption on the hormone estradiol using the Egger test. The results show no publication bias p for bias was 0.871 in Fig. 6d.

**Fig. 7:**
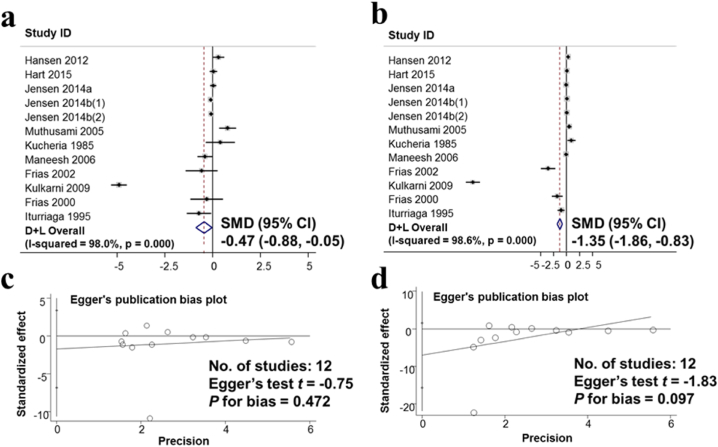
Observed associations between Alcohol consumption and Hormone FSH, Hormone LH. a. Forest plot for the effect of alcohol consumption and hormone FSH; b. Forest plot for the effect of alcohol consumption and hormone LH; c. Evaluation of publication bias about the hormone FSH using Egger publication bias plot; d. Evaluation of publication bias LH using Egger publication bias plot.

Results of the FSH hormone survey in 9375 men from 12 studies and LH hormone in 11,458 men from 12 studies show that the standard means difference in SMD were −0.47; (95% CI: −0.88, −0.05) and −1.35; (95% CI: −1.86, −0.83), respectively. This suggests that drinking alcohol lowers the hormones FSH and LH (Fig. 7a and b). Evaluation of publication bias in publications on the effects of alcohol consumption on hormones FSH and LH by the Egger test. The results showed no publication bias for both FSH or LH with p for bias being 0.472 and 0.097, respectively (Fig. 7c and d).

**Fig. 8:**
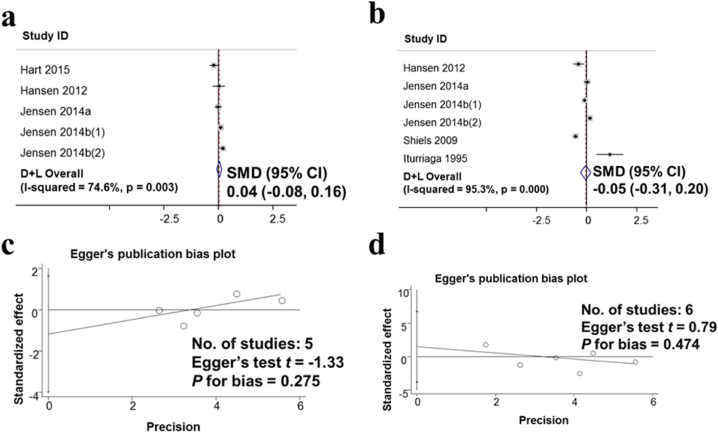
Non-observed associations between Alcohol consumption Hormone Inhibin B, Hormone SHBG2. a. Forest plot for the effect of alcohol consumption and hormone Inhibin B; b. Forest plot for the effect of alcohol consumption and hormone SHBG2; c. Evaluation of publication bias Inhibin B using Egger publication bias plot; d. Evaluation of SHBG2 hormone-related publication bias using Egger publication bias plot.

Inhibin B hormone survey from 5 studies including 10,782 men and SHBG2 hormone from 6 studies including 11,215 men are shown in Fig. 8. The standard means difference SMD for inhibin B was 0.04 (95% CI: −0.08, 0.16); SMD for SHBG2 was −0.05; (95% CI: −0.31, 0.2). Thus, using alcohol has not changed the hormone Inhibin B and SHBG2 (Fig. 8a and b). The assessment of publication bias in the publications on the impact of alcohol consumption on inhibin B and SHBG2 using the Egger test. The results show no publication bias for both inhibin B and SHBG2 with p for bias being 0.275 and 0.474, respectively (Fig. 8c and d).

### 3.5 Subgroup analysis of the associations between alcohol consumption at different levels on male reproductive function

Subgroup analysis was conducted to assess the impact of different levels of alcohol consumption on male fertility. The results presented in Table 2 show that the alcoholic group (drinking at least 1 unit of alcohol per week) had a decrease of semen volume with the SMD was −0.51 (95% CI: −0.77, −0.25); the antioxidant with SMD was −7.93 (95% CI: −12.59, −3.28); testosterone with SMD was −1.60 (95% CI: −2.05, −1.15); FSH with SMD was −0.47 (95% CI: −0.88, 0.05); LH with SMD was −1.35 (95% CI: −1.86, −0.83) compared to the no-alcoholic group. Meanwhile, the moderate alcoholic group (who have 1–7 units of alcohol per week) had no statistical impact on seminal parameters (volume, density, mobility, normal morphology) and sperm abnormalities as well as sex hormones (testosterone, estradiol, FSH, LH, Inhibin B, SHBG2) in comparison to the no-alcoholic group. Comparing the heavy alcoholic group (consuming more than 7 units a week) and the no-alcoholic group, there were significant associations on male reproductive function, likely to reduce the volume of semen per ejaculation with SMD was −0.48 (95% CI: −0.86, −0.11); testosterone with SMD was −1.92 (95% CI: −2.70, −1.13); FSH with SMD was −0.77 (95% CI: −1.47, −0.06), but increased estradiol with SMD was 0.22 (95% CI: 0.00, 0.44); and increased LH with SMD was 0.06= (95% CI: 0.01, 0.12). When analyzing two subgroups of the heavy alcoholic group and the moderate alcoholic group, there was no impact on seminal parameters, but the heavy alcoholic group was shown to maintain a significant association with the increase in estradiol (SMD = 0.54 (95% CI: 0.06, 1.02)) and LH (SMD = 0.22 (95% CI: 0.00, 0.43)) (Table 2).

**Table 2:** Subgroup analysis of the associations between alcohol consumption at different levels on male reproductive function.

Research Index	Comparison between subgroups of alcohol consumption at different levels															
	Alcoholics Vs. No alcoholics				Moderate alcoholics vs. No alcoholics				Heavy alcoholics vs. No alcoholics				Heavy alcoholics vs. Moderate alcoholics			
	n	men	SMD (95% CI)		n	men	SMD (95% CI)		n	men	SMD (95% CI)		n	men	SMD (95% CI)	
Sperm parameter																
Semen volume (ml)	18	17,144	−0.51 (−0.77, −0.25)	↓	6	3148	−0.71 (−1.85, 0.42)	↔	9	6869	−0.48 (−0.86, −0.11)	↓	8	2820	−0.05 (−0.15, 005)	↔
Concentration (10^6^/mL)	23	19,436	0.15 (−0.14, 0.43)	↔	8	3859	−0.02 (−0.18, 0.15)	↔	11	7383	−0.06 (−0.25, 0.12)	↔	10	3641	−0.09 (−0.29, 0.11)	↔
Progressive motility (%)	22	18,549	0.11 (−0.17, 0.40)	↔	7	3276	0.35 (−0.10, 0.79)	↔	10	6959	0.19 (−0.14, 0.52)	↔	9	2874	−0.31 (−0.67, 0.04)	↔
Normal morphology	12	14,296	−0.43 (−1.35, 0.49)	↔	3	1331	−0.08 (−0.28, 0.13)	↔	5	5700	−0.09 (−0.15, −0.03)	↔	5	1154	−0.29 (−0.79, 0.21)	↔
Abnormal morphology	9	2071	0.10 (−0.58, 0.77)	↔	3	1011	0.10 (−0.05, 0.24)	↔	4	1271	−0.22 (−1.21, 0.77)	↔	3	300	0.09 (−0.20, 0.38)	↔
Antioxidant and sperm DNA fragmentation																
Antioxidant	7	1400	7.93 (−12.59, −3.28)	↓	N/A				N/A				N/A			
DNA fragmentation	9	1883	0.59 (−0.17, 1.34)	↔	N/A				N/A				N/A			
Hormone																
Testosterone	17	13,373	−1.60 (−2.05, −1.15)	↓	4	1676	0.05 (−0.09, 0.18)	↔	7	6415	−1.92 (−2.70, −1.13)	↓	5	1981	0.02 (−0.26, 0.29)	↔
Estradiol	8	4143	−0.03 (−0.47, 0.41)	↔	3	1577	0.01 (−0.18, 0.20)	↔	3	855	0.22 (0.00, 0.44)	↑	4	1808	0.54 (0.06, 1.02)	↑
FSH	12	9375	−0.47 (−0.88, 0.05)	↓	3	1577	0.04 (−0.11, 0.20)	↔	6	6208	−0.77 (−1.47, −0.06)	↓	3	1732	0.07 (−0.04, 0.18)	↔
LH	12	11,458	−1.35 (−1.86, −0.83)	↓	3	1577	−0.03 (−0.19, 0.13)	↔	5	5849	0.06 (0.01, 0.12)	↑	4	1808	0.22 (0.00, 0.43)	↑
Inhibin B	5	10,782	0.04 (−0.08, 0.16)	↔	3	1577	−0.07 (−0.22, 0.08)	↔	3	855	−0.09 (−0.24, 0.05)	↔	3	1732	−0.09 (−0.24, 0.05)	↔
SHBG2	6	11,215	−0.05 (−0.31, 0.20)	↔	2	994	−0.01 (−0.17, 0.15)	↔	4	5425	−0.11 (−0.33, 0.11)	↔	2	965	−0.01 (−0.17, 0.15)	↔

n: publication number; men: number of men participating in the study; SMD: Standard mean difference.

↑: uptrend; ↓: downtrend; ↔: trend does not change; N/A: no data available.

## 4 Discussion

In developed countries in Eastern Europe and Central Asia, the burden of disease caused by alcohol accounts for 12.1%. In several developing countries in the Asia Pacific and South America, this rate is estimated at 6.2% [5,71]. This meta-analytical study, which involves 23,258 men in 40 reports on 5 continents, once again confirms the potential danger to male reproductive quality at various concentrations of alcohol in everyday life.

There is a contradiction in conclusions of the effect of alcohol on male reproductive function. Gou et al. in a report on 66 alcoholics, drinking more than 1 year, results showed that the average sperm count, volume, motility, or percentage of sperm was significantly reduced [72]. Stutz et al. concluded that alcohol use could have had detrimental effects on seminal parameters [25]. In contrast, the findings of Govern et al. demonstrated no statistically significant differences in semen volume, sperm concentration, and percentage of motile sperm of daily drinkers [73].

Oxidative stress is implicated as a cause of sperm DNA damage and it can lead to infertility [[74], [75], [76]]. In men, a high ROS ratio leads to sperm DNA damage and sperm DNA fragmentation [77]. Sperm DNA damage has been evaluated as an important attribution of semen quality [78,79], and higher ROS concentrations may also induce autophagy of spermatozoa [80]. Therefore, to be able to diagnose and treat male infertility effectively, it is necessary to evaluate the oxidative status, antioxidant defense system and DNA damage, and sperm parameters [76]. Our study results showed that drinking alcohol reduced antioxidant enzymes in semen, but did not change sperm DNA fragmentation index. In addition, we do not have enough studies to analyze how different levels of alcohol intake affect antioxidant enzymes and sperm DNA fragmentation.

The association between total testosterone levels and alcohol drinking habits was not previously found [62,[81], [82], [83], [84]]. In contrast, reports have shown that men with alcoholism will have a decrease in serum testosterone levels accompanied by a low LH and FSH, which may result from two mechanisms: alcohol destroys Leydig cells or affects the metabolism of hypothalamic-pituitary-gonadal activity [85]. Our results have also shown the effect of alcohol on reproductive hormones, which reduces testosterone, FSH, and LH.

Performing a subgroup analysis provides a clearer view and several interesting results when looking at each group separately. The report also advises that heavy drinkers should be encouraged to cut back [71]. As previous studies only focused on studying two main groups of drinkers and non-drinkers, or heavy drinkers and drinkers, our study expanded the study groups to four subgroups, including the comparison of the alcoholic vs the no-alcoholic group, the moderate alcoholic vs the non-alcoholic groups, the heavy alcoholic vs the no-alcoholic groups, the heavy alcoholic vs the moderate alcoholic groups. This helps our study to determine exactly how alcohol consumption levels affect fertility in men. The heavy alcohol consumption groups have been shown to affect sperm quality, decreasing testosterone and increasing estradiol. Drinking alcohol at all levels does not affect Inhibin B and SHBG2 which is contrary to several previous studies [19,60]. Subgroup analysis offers interesting conclusions, moderate drinking (less than 7 alcohol units per week) has no effects on male fertility. Heavy drinking (more than 7 units/week) negatively impacts male reproductive health.

Around the world, alcohol use among men should be considered an important public health issue. During the five-year period from 2000 to 2005, it was alarmed noting that the rate of alcohol use among Vietnamese people increased rapidly rate by 50% [86,87], besides that the data related to epidemiological data shows that the harmful effects of alcohol use on public health and society are increasing [88].

In conclusion, the meta-analysis of 40 studies surveying 23,258 men showed that alcohol consumption hurt ejaculate semen volume, antioxidant enzymes in semen, and sex hormone levels. However, drinking alcohol at a moderate level (less than 7 alcohol units per week) is unlikely to change the parameters of semen and sex hormones, while drinking at a heavy level (more than 7 units/per week) is likely to make negative effects on semen volume and sex hormones.

## Author contribution statement

Tung Nguyen-Thanh, Ph.D.: Conceived and designed the experiments; Performed the experiments; Analyzed and interpreted the data; Contributed reagents, materials, analysis tools or data; Wrote the paper.

Ai-Phuong Hoang-Thi: Performed the experiments; Analyzed and interpreted the data; Contributed reagents, materials, analysis tools or data; Wrote the paper.

Dang Thi Anh Thu: Analyzed and interpreted the data; Contributed reagents, materials, analysis tools or data; Wrote the paper.

## Data availability statement

Data included in article/supp. material/referenced in article.

## Declaration of interest’s statement

The authors declare that they have no known competing financial interests or personal relationships that could have appeared to influence the work reported in this paper.

## Additional information

No additional information is available for this paper.

## References

[1] Heath D.B. (2012).

[2] Room R., Babor T., Rehm J. (2005). Alcohol and public health. Lancet.

[3] Rehm J. (2009). Global burden of disease and injury and economic cost attributable to alcohol use and alcohol-use disorders. Lancet.

[4] Organization W.H. (2019).

[5] Giang K.B. (2008). Alcohol use and alcohol consumption–related problems in rural Vietnam: an epidemiological survey using AUDIT. Subst. Use Misuse.

[6] Murray C.J., Lopez A.D. (1997). Global mortality, disability, and the contribution of risk factors: global Burden of Disease Study. Lancet.

[7] Single E. (1999). Morbidity and mortality attributable to alcohol, tobacco, and illicit drug use in Canada. Am. J. Publ. Health.

[8] Gutjahr E., Gmel G., Rehm J. (2001). Relation between average alcohol consumption and disease: an overview. Eur. Addiction Res..

[9] Rehm J. (2003). The global distribution of average volume of alcohol consumption and patterns of drinking. Eur. Addiction Res..

[10] Goodlett C.R., Horn K.H. (2001). Mechanisms of alcohol-induced damage to the developing nervous system. Alcohol Res. Health.

[11] Ji C. (2015). Advances and new concepts in alcohol-induced organelle stress, unfolded protein responses and organ damage. Biomolecules.

[12] Obad A. (2018). Alcohol-mediated organ damages: heart and brain. Front. Pharmacol..

[13] Thomes P.G. (2021). Natural recovery by the liver and other organs after chronic alcohol use. Alcohol Res. Curr. Rev..

[14] Manzo-Avalos S., Saavedra-Molina A. (2010). Cellular and mitochondrial effects of alcohol consumption. Int. J. Environ. Res. Publ. Health.

[15] Hansen M.L. (2012). Does last week's alcohol intake affect semen quality or reproductive hormones? A cross-sectional study among healthy young Danish men. Reprod. Toxicol..

[16] Pajarinen J. (1996). Moderate alcohol consumption and disorders of human spermatogenesis. Alcohol Clin. Exp. Res..

[17] Emanuele M.A., Emanuele N.V. (1998). Alcohol's effects on male reproduction. Alcohol Health Res. World.

[18] Jensen T.K. (2014). Habitual alcohol consumption associated with reduced semen quality and changes in reproductive hormones; a cross-sectional study among 1221 young Danish men. BMJ Open.

[19] Jensen T.K. (2014). Alcohol and male reproductive health: a cross-sectional study of 8344 healthy men from Europe and the USA. Hum. Reprod..

[20] Li Y. (2011). Association between socio-psycho-behavioral factors and male semen quality: systematic review and meta-analyses. Fertil. Steril..

[21] Ricci E. (2017). Semen quality and alcohol intake: a systematic review and meta-analysis. Reprod. Biomed. Online.

[22] Gaur D.S., Talekar M.S., Pathak V.P. (2010). Alcohol intake and cigarette smoking: impact of two major lifestyle factors on male fertility. Indian J. Pathol. Microbiol..

[23] Martini A.C. (2004). Effects of alcohol and cigarette consumption on human seminal quality. Fertil. Steril..

[24] Muthusami K., Chinnaswamy P. (2005). Effect of chronic alcoholism on male fertility hormones and semen quality. Fertil. Steril..

[25] Stutz G. (2004). The effect of alcohol, tobacco, and aspirin consumption on seminal quality among healthy young men. Arch. Environ. Health.

[26] Aboulmaouahib S. (2018). Impact of alcohol and cigarette smoking consumption in male fertility potential: looks at lipid peroxidation, enzymatic antioxidant activities and sperm DNA damage. Andrologia.

[27] Zini A., San Gabriel M., Baazeem A. (2009). Antioxidants and sperm DNA damage: a clinical perspective. J. Assist. Reprod. Genet..

[28] Barratt C.L. (2017). The diagnosis of male infertility: an analysis of the evidence to support the development of global WHO guidance—challenges and future research opportunities. Hum. Reprod. Update.

[29] Teijon M.L. (2007). Semen quality in a population of volunteers from the province of Barcelona. Reprod. Biomed. Online.

[30] Luo D. (2018). Optimally estimating the sample mean from the sample size, median, mid-range, and/or mid-quartile range. Stat. Methods Med. Res..

[31] Wan X. (2014). Estimating the sample mean and standard deviation from the sample size, median, range and/or interquartile range. BMC Med. Res. Methodol..

[32] Hozo S.P., Djulbegovic B., Hozo I. (2005). Estimating the mean and variance from the median, range, and the size of a sample. BMC Med. Res. Methodol..

[33] Brennan A. (2014). Potential benefits of minimum unit pricing for alcohol versus a ban on below cost selling in England 2014: modelling study. BMJ.

[34] Anifandis G. (2014). The impact of cigarette smoking and alcohol consumption on sperm parameters and sperm DNA fragmentation (SDF) measured by Halosperm((R)). Arch. Gynecol. Obstet..

[35] Chia S.E., Tay S.K., Lim S.T. (1998). What constitutes a normal seminal analysis? Semen parameters of 243 fertile men. Hum. Reprod..

[36] Condorelli R.A. (2015). Chronic consumption of alcohol and sperm parameters: our experience and the main evidences. Andrologia.

[37] Eskenazi B. (2003). The association of age and semen quality in healthy men. Hum. Reprod..

[38] Goverde H.J. (1995). Semen quality and frequency of smoking and alcohol consumption--an explorative study. Int. J. Fertil. Menopausal Stud..

[39] Hart R.J. (2015). Testicular function in a birth cohort of young men. Hum. Reprod..

[40] Jensen T.K. (2014). Alcohol and male reproductive health: a cross-sectional study of 8344 healthy men from Europe and the USA. Hum. Reprod..

[41] Joo K.J. (2012). The effects of smoking and alcohol intake on sperm quality: light and transmission electron microscopy findings. J. Int. Med. Res..

[42] Kumar S. (2014). Environmental & lifestyle factors in deterioration of male reproductive health. Indian J. Med. Res..

[43] Lopez Teijon M. (2007). Semen quality in a population of volunteers from the province of Barcelona. Reprod. Biomed. Online.

[44] Martini A.C. (2004). Effects of alcohol and cigarette consumption on human seminal quality. Fertil. Steril..

[45] Muthusami K.R., Chinnaswamy P. (2005). Effect of chronic alcoholism on male fertility hormones and semen quality. Fertil. Steril..

[46] Kucheria K., Saxena R., Mohan D. (1985). Semen analysis in alcohol dependence syndrome. Andrologia.

[47] Brzek A. (1987). Alcohol and male fertility (preliminary report). Andrologia.

[48] de Jong A.M. (2014). Effect of alcohol intake and cigarette smoking on sperm parameters and pregnancy. Andrologia.

[49] Komiya A. (2014). Clinical factors associated with sperm DNA fragmentation in male patients with infertility. Sci. World J..

[50] Keskin M.Z. (2016). Do cigarette and alcohol affect semen analysis?. Arch. Ital. Urol. Androl..

[51] Wdowiak A. (2016). Relationship between alcohol consumption and sperm nuclear DNA fragmentation and pregnancy POST ĘP. Y ANDROLOGI I ONLINE.

[52] Ricci E. (2018). Alcohol intake and semen variables: cross-sectional analysis of a prospective cohort study of men referring to an Italian Fertility Clinic. Andrology.

[53] Schmid T.E. (2007). The effects of male age on sperm DNA damage in healthy non-smokers. Hum. Reprod..

[54] Varshini J. (2012). Poor sperm quality and advancing age are associated with increased sperm DNA damage in infertile men. Andrologia.

[55] Schmid T.E. (2012). Micronutrients intake is associated with improved sperm DNA quality in older men. Fertil. Steril..

[56] Marshburn P.B., Sloan C.S., Hammond M.G. (1989). Semen quality and association with coffee drinking, cigarette smoking, and ethanol consumption. Fertil. Steril..

[57] Wogatzky J. (2012). The combination matters--distinct impact of lifestyle factors on sperm quality: a study on semen analysis of 1683 patients according to MSOME criteria. Reprod. Biol. Endocrinol..

[58] Gautam S. (2015). Sperm DNA damage in non-familial sporadic heritable retinoblastoma (NFSHRb). Clin. Epidemiol. Global Health.

[59] Maneesh M. (2006). Alcohol abuse-duration dependent decrease in plasma testosterone and antioxidants in males. Indian J. Physiol. Pharmacol..

[60] Shiels M.S. (2009). Association of cigarette smoking, alcohol consumption, and physical activity with sex steroid hormone levels in US men. Cancer Causes Control.

[61] Frias J. (2002). Effects of acute alcohol intoxication on pituitary-gonadal axis hormones, pituitary-adrenal axis hormones, beta-endorphin and prolactin in human adults of both sexes. Alcohol Alcohol.

[62] Handa K. (1997). Behavioral correlates of plasma sex hormones and their relationships with plasma lipids and lipoproteins in Japanese men. Atherosclerosis.

[63] Kulkarni S.R. (2009). Levels of plasma testosterone, antioxidants and oxidative stress in alcoholic patients attending de-addiction centre. Biol. Med..

[64] Oldereid N.B., Rui H., Purvis K. (1992). Lifestyles of men in barren couples and their relationships to sperm quality. Eur. J. Obstet. Gynecol. Reprod. Biol..

[65] Close C.E., Roberts P.L., Berger R.E. (1990). Cigarettes, alcohol and marijuana are related to pyospermia in infertile men. J. Urol..

[66] von der P.B. (2002). Testosterone, 5 alpha-dihydrotestosterone and cortisol in men with and without alcohol-related aggression. J. Stud. Alcohol.

[67] Sierksma A. (2004). Effect of moderate alcohol consumption on plasma dehydroepiandrosterone sulfate, testosterone, and estradiol levels in middle-aged men and postmenopausal women: a diet-controlled intervention study. Alcohol Clin. Exp. Res..

[68] Frias J. (2000). Effects of acute alcohol intoxication on pituitary-gonadal axis hormones, pituitary-adrenal axis hormones, beta-endorphin and prolactin in human adolescents of both sexes. Life Sci..

[69] Iturriaga H. (1995). Effects of abstinence on sex hormone profile in alcoholic patients without liver failure. J. Endocrinol. Invest..

[70] Dai W.S. (1981). The epidemiology of plasma testosterone levels in middle-aged men. Am. J. Epidemiol..

[71] Di Castelnuovo A. (2006). Alcohol dosing and total mortality in men and women: an updated meta-analysis of 34 prospective studies. Arch. Intern. Med..

[72] Guo H. (2006). Effects of cigarette, alcohol consumption and sauna on sperm morphology. Zhonghua nan ke xue= Natl. J. Androl..

[73] Goverde H.J. (1995).

[74] Aitken R.J. (2014). Oxidative stress and male reproductive health. Asian J. Androl..

[75] Bisht S., Dada R. (2017). Oxidative stress: major executioner in disease pathology, role in sperm DNA damage and preventive strategies. Front. Biosci.-Scholar.

[76] Hosen M.B. (2015). Oxidative stress induced sperm DNA damage, a possible reason for male infertility. Iran. J. Reproductive Med..

[77] Makker K., Agarwal A., Sharma R. (2009). Oxidative stress & male infertility. Indian J. Med. Res..

[78] Aitken R.J. (2010). Analysis of the relationships between oxidative stress, DNA damage and sperm vitality in a patient population: development of diagnostic criteria. Hum. Reprod..

[79] Lewis S.E. (2013). The impact of sperm DNA damage in assisted conception and beyond: recent advances in diagnosis and treatment. Reprod. Biomed. Online.

[80] Aitken R.J., Curry B.J. (2011). Redox regulation of human sperm function: from the physiological control of sperm capacitation to the etiology of infertility and DNA damage in the germ line. Antioxidants Redox Signal..

[81] Allen N.E. (2002). Lifestyle and nutritional determinants of bioavailable androgens and related hormones in British men. Cancer Causes Control.

[82] Medina D.L., Santisteban P. (2000).

[83] Svartberg J., Midtby M., Bonaa K.H., Sundsfjord J., Joakimsen R.M., Jorde R. (2003). The associations of age, lifestyle factors and chronic disease with testosterone in men: the Tromso Study. Eur. J. Endocrinol..

[84] Hsieh C.-c. (1998). Predictors of sex hormone levels among the elderly: a study in Greece. J. Clin. Epidemiol..

[85] Maneesh M. (2006). Alcohol abuse-duration dependent decrease in plasma testosterone and antioxidants in males. Indian J. Physiol. Pharmacol..

[86] Bao Giang K., Van Minh H., Allebeck P. (2013). Alcohol consumption and household expenditure on alcohol in a rural district in Vietnam. Glob. Health Action.

[87] Poznyak V. (2013). The world health organization's global monitoring system on alcohol and health. Alcohol Res. Curr. Rev..

[88] Lincoln M. (2016). Alcohol and drinking cultures in Vietnam: a review. Drug Alcohol Depend..

